# Stability of healthcare quality measures for maternal and child services: Analysis of the continuous service provision assessment of health facilities in Senegal, 2012–2018

**DOI:** 10.1111/tmi.13701

**Published:** 2021-12-16

**Authors:** Hannah H. Leslie, Celestin Hategeka, Papa Ibrahima Ndour, Kojo Nimako, Mamadou Dieng, Abdoulaye Diallo, Youssoupha Ndiaye

**Affiliations:** ^1^ Division of Prevention Science Department of Medicine University of California San Francisco California USA; ^2^ Department of Global Health and Population Harvard TH Chan School of Public Health Boston Massachusetts USA; ^3^ Directorate of Planning, Research, and Statistics Ministry of Health and Social Action Dakar Senegal; ^4^ Agence Nationale de la Démographie et de la Statistique Dakar Senegal; ^5^ Health Systems Consultant Accra Ghana

**Keywords:** child health, health services, healthcare quality, maternal health, Senegal

## Abstract

**Objective:**

High‐quality healthcare is essential to ensuring maternal and newborn survival. Efficient measurement requires knowing how long measures of quality provide consistent insight for intended uses.

**Methods:**

We used a repeated health facility assessment in Senegal to calculate structural and process quality of antenatal care (ANC), delivery and child health services in facilities assessed 2 years apart. We tested agreement of quality measures within facilities and regions. We estimated how much input‐adjusted and process quality‐adjusted coverage measures changed for each service when calculated using quality measurements from the same facilities measured 2 years apart.

**Results:**

Over 6 waves of continuous surveys, 628 paired assessments were completed. Changes at the facility level were substantial and often positive, but inconsistent. Structural quality measures were moderately correlated (0.40–0.69) within facilities over time, more so in hospitals; correlation was <0.20 for process measures based on direct observation of ANC and child visits. Most measures were more strongly correlated once averaged to regions; process quality of child services was not (−0.32). Median relative difference in national‐adjusted coverage estimates was 6.0%; differences in subnational estimates were largest for process quality of child services (19.6%).

**Conclusion:**

Continuous measures of structural quality demonstrated consistency at regional levels and in higher level facilities over 2 years; results for process measures were mixed. Direct observation of child visits provided inconsistent measures over time. For other measures, linking population data with health facility assessments from up to 2 years prior is likely to introduce modest measurement error in adjusted coverage estimates.

## INTRODUCTION

The premise of universal health coverage (UHC), enshrined in the Sustainable Development Goals and adopted as national policy by many countries, is essential health service delivery with financial protection. Health systems must be high quality for service coverage to yield better population health outcomes [1, 2]. Given the many demands on health systems even before the ongoing Covid‐19 pandemic, measurement of quality to monitor service delivery and inform health system interventions should be streamlined to optimise utility for policy while minimising cost and burden [3]. For instance, information intended to prompt immediate action such as medication stock‐out should be assessed and transmitted in real time; data intended for periodic benchmarking and comparison should reliably distinguish between levels of quality for the timespan of intended use [4]. Unfortunately, quality measurement at present is fragmented, with use of assessment tools poorly fit for purpose [5, 6].

Health facility assessments are infrequent but typically detailed surveys of service availability, readiness and quality of care that can provide unique value beyond more frequent methods such as routine health information systems [7]. In low‐ and middle‐income countries, such assessments have been used to benchmark health service availability and readiness [8], to compare quality of care across countries [9], to identify better and worse performers [10] and increasingly in assessment of effective coverage (the “fraction of potential health gain actually delivered through the health system to the population in need” [11]) as a composite metric of health system performance [12, 13, 14]. These uses often extend to years past the date of data collection. In particular, estimates of effective coverage may be calculated from health facility assessments and population data measured at different points in time; limited empirical evidence is available to assess the longevity of information from health facility assessments to support this practice [15]. Use of quality measures for description and benchmarking, on their own or as part of effective coverage measures, requires stability in the actual value of the measure from time of measurement to when inference is made; use for identification of better or worse performers relies on stability in classifications over time. If such information is relatively consistent over time, health facility assessments conducted sporadically would remain useful. In contrast, instability in such measures would imply that the data must be used rapidly for it to have value. Stability assessments testing the consistency of a measure over repeated application have been conducted for individual and facility‐level quality of care metrics in the United States [16, 17, 18, 19]. A recent study in South Africa found that laboratory measures of HIV care outcomes showed fairly high reliability year to year across all facility types [20]. Similar methods have yet to be applied to nationally representative health facility assessments.

Measurement stability may vary by type of quality measure. Health facility assessments typically produce measures of structure, or inputs to care, and those with clinical observations also generate measures of processes of care [21, 22]. While the process of care is recognised as more proximal to patient outcomes than inputs alone [5, 23, 24], measuring processes is more complex than itemising inputs. Direct observation requires selection and observation of patients, resulting in sampling error and potentially observer bias [25, 26]. Given calls to increase the use of process measures [5, 6, 24], understanding the stability of both types of measures can inform assessment approaches.

The state of health system measurement in Senegal provides a unique opportunity to assess stability of quality measures. A health facility assessment has been conducted annually in Senegal since late 2012, capturing standardised information on a representative sample of the entire health system. At the same time, the government of Senegal has embedded the steps towards UHC in the *Plan National de Développement Sanitaire et Social* (PNDSS) 2019–2028 [27, 28] and is actively working to take a health systems approach, including a shift away from donor‐supported community campaigns towards facility‐based services [29]. Maternal and child health services are a priority, with efforts to improve newborn and under‐5 survival emphasised given limited gains in these metrics since 2013 [27].

The aims of this analysis are to define structural and process measures relevant to quality of care for maternal and child health across the continuous SPA surveys, to assess stability of such measures at the facility and sub‐national levels based on assessments conducted 2 years apart and to quantify the effect of any instability on effective coverage measures.

## METHODS

### Data sources

We used data from the Service Provision Assessment (SPA), a standardised cross‐sectional survey designed to measure health service availability, readiness and quality [30]. The SPA is implemented in Senegal by the National Statistics and Demographics Agency (ANSD in French) and the Demographic and Health Surveys (DHS) Program [31]. We pooled data from 6 surveys conducted from 2012/2013 to 2018(Record checklist available in Appendix 1).

We used the household surveys conducted annually by ANSD and the DHS Program from 2015 to 2019 to define the populations in need and utilisation of care as part of the calculations of quality‐adjusted coverage [32].

### Sampling

Health services in Senegal are organised within the 14 regions and are offered by hospitals, health centres, health posts staffed by 2 salaried providers and health huts staffed by volunteer community health workers [29]. For the continuous SPA, a master facility list including 1578 hospitals, health centres and health posts was used to select a nationally representative sample of facilities stratified by region and facility type for each of the first 4 years of the survey. Sampling fractions were 50% for hospitals and health centres and 20% for health posts each year; hospitals and health centres not sampled in years 1 and 3 would be sampled in years 2 and 4. Survey implementers thus aimed to resample 100% of hospitals and health centres as well as 30% of the health posts every other year. In 2017, the sampling frame was updated, and subsequent samples were drawn independently. ANSD provided the sampling frames with facility IDs to identify facilities assessed more than once. For 2017, only facility name was available: some facilities assessed in this year may not have been linked due to differences in recorded facility name. Our analytic dataset consists of all pairs of assessments matched for the same facility 2 years apart. We used the classification from the sampling frame in 2 cases where facility tier conflicted in the survey data.

Patients were selected for observation using systematic random sampling from a list of those presenting for services on the day of the visit. Survey data include sampling weights for each facility, provider and client visit.

The DHS household surveys used multistage sampling, first selecting census districts by probability proportional to size and then households with systematic random sampling. Women 15 to 49 years old were approached and interviewed [33]. These surveys are intended to provide representative estimates within 4 major sub‐national regions: West (Dakar and Thiès regions), Centre (Diourbel, Fatick, Kaffrine, Kaolack regions), North (Louga, Matam, and Saint‐Louis regions) and South (Kédogou, Kolda, Sédhiou, Tambacounda, and Ziguinchor regions).

### Measures

We included SPA data from the facility audit of service availability and readiness, from provider interviews, and from direct observations of curative consultations for children under 5 years (all survey waves) as well as of antenatal care (ANC) visits (conducted only during 2014, 2016, and 2018 surveys). We extracted covariates to characterise the sample and consider continuity of providers within facilities over time: location, facility tier, ownership type, number and tenure of staff providing care for the maternal and child health (MCH) services of interest – ANC, delivery and/or curative care for sick children – and number of observations conducted during the assessment. Provider interview data were weighed using within‐facility sampling weights in summarising to facilities.

We analysed measures of structural and process quality for maternal and child health services. For structural quality, we defined service readiness scores based on the WHO Service Availability and Readiness Assessment [34] for general services (48 items) and for ANC, basic obstetric care and care for children (9, 19 and 18 items respectively; Table S1). These scores cover core domains of readiness: infrastructure, basic equipment, infection prevention, diagnostics and essential medications for general service readiness, and staff and guidelines, basic equipment, diagnostics (as applicable) and medications for each service.

For process quality, we defined adherence to global guidelines: the 2016 WHO‐focused ANC model [35] and the 2014 chart book for Integrated Management of Childhood Illness [36]. Visits were scored as the proportion of actions completed out of items assessed (27 for ANC and 25 for child visits, Table S1; actions in ANC follow‐up visits were weighted 1/3, 2/3 or 1 based on recommended frequency for ANC visits 2 through 4). Facilities were assigned the average score of visits observed for each service, weighted by the within‐facility sampling weight. For delivery care, we used proportion of the 7 signal functions for Basic Emergency Obstetric and Newborn Care (BEmONC) reported as performed in the prior 3 months. We considered each quality measure as a continuous score from 0 to 1. To assess stability of classifications of better, average and worse performers, we classified scores into tertiles of the observed range separately for each year of the paired assessments of the same facilities.

To assess stability at the subnational level, we used the 4 major regions defined by the DHS survey (West, Centre, North, South). We averaged scores based on all facilities or all direct observations of care in each of the major regions within the analytic sample of paired assessments of the same facilities, yielding 4 sets of regional measures (2012/2013 with 2015, 2014 with 2016, 2015 with 2017 and 2016 with 2018). Observations were weighted based on the facility or client weight when averaging.

To test the impact of instability in quality measures on effective coverage, we considered 2 steps of the effective coverage cascade: input‐adjusted coverage (“the proportion of the population in need who come into contact with a health service that is ready to provide care”) based on structural quality measures and quality‐adjusted coverage (“the proportion of the population in need who come into contact with a service that is ready and that receives the service according to quality‐of‐care standards” [24]) based on process quality measures. We first defined contact coverage from the DHS surveys for ANC 1 and 4, delivery and child health. For maternal health, the population in need was women with a live birth in the past 2 years, while for child health, this was children under 5 with fever, diarrhoea or respiratory symptoms in the past 2 weeks. Use of care was reported use of a hospital, health centre or health post (minimum 1 and 4 visits for ANC 1 and 4, respectively). We grouped reported facility type as public hospital, public health centre, public health post and any private facility to enable linkage to SPA data; women reporting multiple sources of ANC were assigned the highest reported facility type in the order listed. Because the analytic sample includes only these facility types, use of health huts or unclassified facilities was defined as not receiving services. The resulting estimates are intended only to inform consideration of measurement stability over time.

We calculated adjusted coverage as the product of contact coverage and quality. We defined the reference period for health facility assessments as 1 year prior for maternal health (women interviewed in 2018 regarding pregnancies in the past 2 years would be matched to the 2017 SPA data) and in the same year for child health. We calculated adjusted coverage nationally and within major region, accounting for type of facility and incorporating stratified sampling and survey weights (women's weight in DHS data, facility weight in SPA data) following established procedures [37, 38]. Adjusted coverage measures are based on sample facilities assessed during the reference period and again using the same facilities assessed 2 years earlier. We limited analysis to estimates for regions that had at least one facility of each type that DHS respondents reported using.

### Analysis

In this analysis, we (1) describe the analytic sample of paired assessments to explain the basis of analysis and how it differs from a full SPA and (2) assess stability over 2 years for facilities, regions and in adjusted coverage estimation. We used descriptive statistics to summarise the pooled sample of hospitals, health centres and health posts assessed between 2012/2013 and 2018. We compared facilities included in the repeated assessment to those excluded using descriptive statistics and, for continuous characteristics, ANOVA tests incorporating clustering due to repeated inclusion of facilities assessed three times. This comparison is unweighted; pooled data and the analytic sample of repeated facilities are not representative of a predefined target population.

To define stability of quality measures for description and benchmarking, we assessed continuous quality measures. We quantified the magnitude of change in quality measures as the absolute value of the difference between time points for each facility; to capture overall (net) difference, we averaged observed difference by facility tier and by region. We calculated Pearson correlation coefficients for quality scores for facilities and regions at measured 2 years apart (negligible <0.10, weak 0.10–0.39, moderate 0.40–0.69, strong 0.70–0.89 and very strong 0.90–1.00) [39]. Facility‐level correlations are plotted, with random jitter added for illustration purposes for ANC readiness and proportion of BEmONC signal functions. An overlaid ellipse outlines where 80% of the data would lie assuming bivariate normality and signals degree of correlation: a circle indicates no correlation, a tighter diagonal ellipse high correlation. Facility‐level correlation statistics are weighted by the facility sampling weight from the earlier wave of each pair; we conducted sensitivity analysis using the weights from the later wave.

To quantify the stability of quality measures used to identify relatively better or worse performing facilities, we calculated percent agreement and Cohen's kappa for categorical measures.

To assess the impact of instability in quality measures on adjusted coverage of health services, we calculated differences for each adjusted coverage metric (detailed in Table S2). We plotted and summarised differences for national and sub‐national estimates resulting from using measures of the same facilities 2 years before the reference period.

Analyses were conducted in R and Stata.

### Ethics statement

The Harvard University Human Research Protection Program approved this secondary analysis as exempt; the original survey implementers obtained ethical approvals for data collection.

### Patient and public involvement

It was not appropriate to involve patients or the public in the design, or conduct, or reporting or dissemination plans of this research.

## RESULTS

### Analytic sample

**FIGURE 1:**
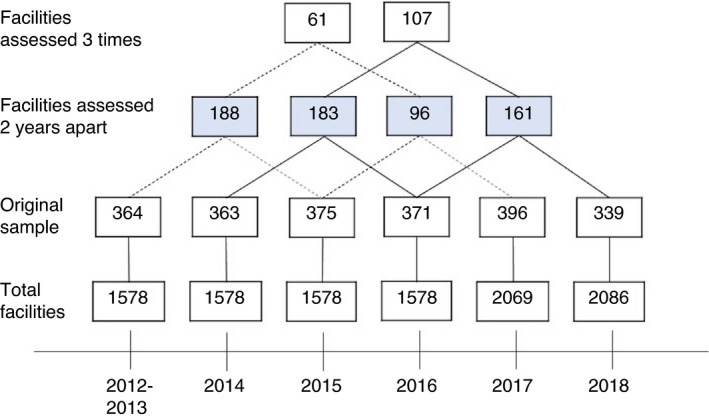
Schematic of SPA sampling and creation of analytic dataset

From the 2012/2013 survey through 2018, 16% to 24% of hospitals, health centres, and health posts on the master facility list were successfully assessed, leading to 2208 completed assessments (Figure 1, Table S3). We identified 628 paired assessments of the same facilities that form the analytic sample.

Table 1 compares the characteristics of the 1120 assessments that took place in facilities sampled once to the 1088 assessments in the 460 facilities sampled more than once. The overrepresentation of higher level facilities in the resampling approach results in a sample of facilities that are larger and more urban, with more providers and visits observed for ANC and curative care for children, and higher service readiness and performance of BEmONC signal functions. The average tenure of providers is comparable and the proportion of providers assigned directly is higher, suggesting that retention of providers over 2 years should be at least as high in the repeated sample as the facilities assessed once.

**TABLE 1:** Characteristics of assessments conducted in facilities assessed one time and those assessed repeatedly

	One‐time	Repeated	Total
	(*N* = 1120)	(*N* = 1088)	(*N* = 2208)
Facility location			
Rural	622 (56%)	423 (39%)	1045 (47%)
Urban	498 (44%)	665 (61%)	1163 (53%)
Facility type			
Hospital	34 (3%)	173 (16%)	207 (9%)
Health centre	53 (5%)	335 (31%)	388 (18%)
Health post	1033 (92%)	580 (53%)	1613 (73%)
Facility managing authority			
Public	922 (82%)	848 (78%)	1770 (80%)
Private	198 (18%)	240 (22%)	438 (20%)
4 major regions			
West	273 (24%)	350 (32%)	623 (28%)
Centre	294 (26%)	231 (21%)	525 (24%)
North	224 (20%)	185 (17%)	409 (19%)
South	329 (29%)	322 (30%)	651 (29%)

	One‐time	Repeated	Total	
	(*N* = 1120)	(*N* = 1088)	(*N* = 2208)	
	Mean ± SD	Mean ± SD	Mean ± SD	*p*‐value
Facility characteristics				
MCH staff				
*N*	3.07 ± 2.02	4.39 ± 3.85	3.72 ± 3.13	<0.001
Tenure in facility	7.55 ± 5.75	7.31 ± 5.54	7.43 ± 5.65	0.597
Proportion with tenure >2 years	0.66 ± 0.34	0.64 ± 0.32	0.65 ± 0.33	0.195
Proportion assigned directly to facility	0.58 ± 0.35	0.63 ± 0.34	0.61 ± 0.34	0.013
Observed in ANC visits (*N* = 796 facility assessments)	1.02 ± 0.15	1.10 ± 0.34	1.06 ± 0.27	<0.001
Observed in child visits (*N* = 1827 facility assessments)	1.03 ± 0.18	1.08 ± 0.31	1.06 ± 0.26	<0.001
Observations				
ANC visits (*N* = 796 facility assessments)	2.94 ± 1.76	3.59 ± 2.12	3.29 ± 1.99	<0.001
Child visits (*N* = 1827 facility assessments)	3.32 ± 1.74	3.89 ± 1.97	3.60 ± 1.88	<0.001
Quality measures				
Service readiness: general	0.64 ± 0.11	0.67 ± 0.12	0.66 ± 0.11	<0.001
Service readiness: antenatal care	0.72 ± 0.14	0.73 ± 0.15	0.72 ± 0.15	0.036
Service readiness: basic obstetric care	0.60 ± 0.17	0.63 ± 0.17	0.61 ± 0.17	<0.001
Service readiness: preventive & curative care for children	0.65 ± 0.17	0.65 ± 0.19	0.65 ± 0.18	0.410
Adherence to guidelines, antenatal care	0.61 ± 0.13	0.60 ± 0.12	0.60 ± 0.12	0.809
Proportion of BEmONC signal functions (out of 7)	0.70 ± 0.20	0.76 ± 0.21	0.73 ± 0.21	<0.001
Adherence to guidelines, curative care for children <5	0.37 ± 0.12	0.36 ± 0.12	0.36 ± 0.12	0.342

Abbreviations: BEmONC, Basic Emergency Obstetric and Newborn Care; MCH, Maternal and child health; SD, Standard deviation.

Table 2 details the analytic sample of the 628 paired assessments by facility type and quality measure. The sample size is smallest for process quality of ANC (228 paired assessments). A median of five child visits and 3 to 4 ANC visits were observed across the sample; fewer visits were observed in health posts than health centres and hospitals, and the number of visits declined in the second wave of each paired assessment. A median of 1 provider was observed for each service and all facility types (data not shown).

**TABLE 2:** Number of facilities and number of visits in analytic sample

	Hospital	Health centre	Health post	Total
	(*N* = 104)	(*N* = 211)	(*N* = 313)	(*N* = 628)
Facilities with matched assessments by quality measure				
Service readiness: general	104	211	313	628
Service readiness: antenatal care	83	164	275	522
Service readiness: basic obstetric care	85	147	251	483
Service readiness: preventive & curative care for children	87	198	298	583
Adherence to guidelines, antenatal care	33	82	113	228
Proportion of BEmONC signal functions	85	147	251	483
Adherence to guidelines, curative care for children	66	175	252	493

	Hospital	Health centre	Health post	Total
Visits observed	Median (IQR)	Median (IQR)	Median (IQR)	Median (IQR)
Antenatal care, wave 1	5.0 (3.0, 5.0)	5.0 (4.0, 5.0)	3.0 (2.0, 5.0)	4.0 (2.0, 5.0)
Antenatal care, wave 2	3.0 (2.0, 5.0)	4.0 (2.0, 5.0)	2.0 (1.0, 5.0)	3.0 (2.0, 5.0)
Curative care for children, wave 1	5.0 (5.0, 5.0)	5.0 (4.0, 5.0)	4.0 (2.0, 5.0)	5.0 (3.0, 5.0)
Curative care for children, wave 2	5.0 (4.0, 5.0)	5.0 (3.0, 5.0)	3.0 (2.0, 5.0)	5.0 (2.0, 5.0)

### Stability of quality measures within facilities

**FIGURE 2:**
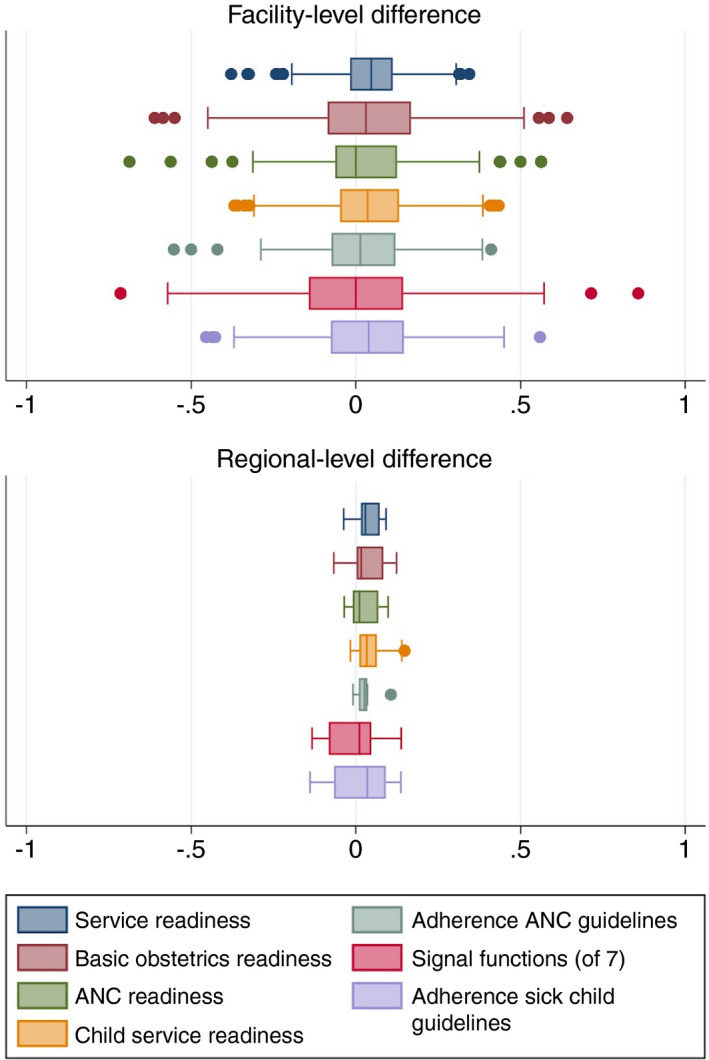
Difference in quality measures between time points

Individual facilities changed substantially for each measure over 2 years: the absolute value of the difference for a given facility over 2 years ranged from 0.08 (general service readiness) to 0.18 (BEmONC signal functions) on average, with some facilities changing as much as ±0.50 out of 1.0 (Figure 2). The magnitude of difference was generally similar across health facility types (Table S4). Net change was generally positive and modest: 0.01 to 0.05 linear difference on average.

**FIGURE 3:**
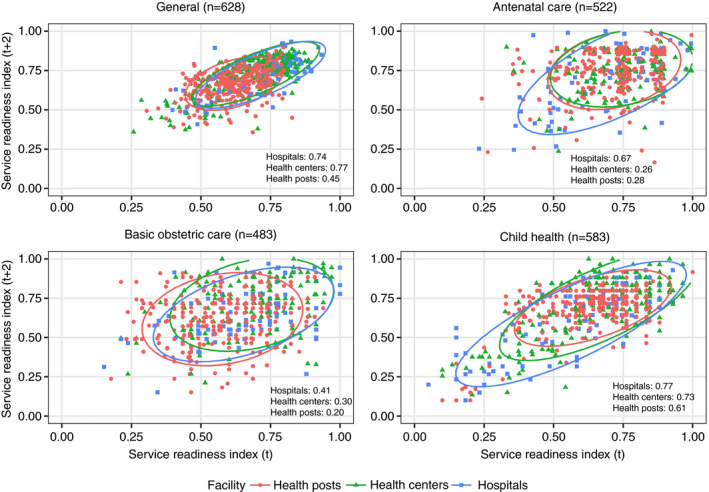
Correlation of structural quality measures for individual facilities measured 2 years apart

Structural quality measures were weakly to moderately correlated over 2 years, more strongly so in higher level facilities and particularly for general readiness and readiness for child care services (Figure 3). Across all facilities, correlation was moderate for these measures but weak for ANC readiness and basic obstetric readiness (Table 3A). Correlation for general service readiness was driven largely by domains of basic amenities (correlation 0.68) and medications, particularly in hospitals (correlation 0.56, 0.70 in hospitals), while basic equipment and infection prevention domains were less correlated (Table S5). Readiness was less correlated for ANC and basic obstetrics compared to child services within each domain, with the largest difference – correlation of 0.0 for equipment in ANC compared to over 0.50 in the other services – notably in a domain where there is only 1 item for ANC.

**TABLE 3:** Correlation of quality of care over 2 years

A. Facilities	Total observations	Correlation	Percent agreement (tertiles)	Cohen's Kappa (tertiles)
Structural quality measures				
General	628	0.60	55%	0.33
Antenatal care	522	0.34	46%	0.18
Basic obstetrics	483	0.28	45%	0.18
Care for children	583	0.68	51%	0.26
Process quality measures				
Antenatal care	228	0.24	37%	0.06
Basic obstetrics	483	0.35	53%	0.25
Care for children	493	0.05	36%	0.04

B. Regions	*N*	Correlation	Facilities per region Median	Visits observed per region Median
Structural quality measures				
General	16	0.52	36.5	NA
Antenatal care	16	0.57	32.5	NA
Basic obstetrics	16	0.67	31.0	NA
Care for children	16	0.86	34.0	NA
Process quality measures				
Antenatal care	8	0.75	28.0	111.0
Basic obstetrics	16	0.26	31.0	NA
Care for children	16	−0.36	29.5	108.3

**FIGURE 4:**
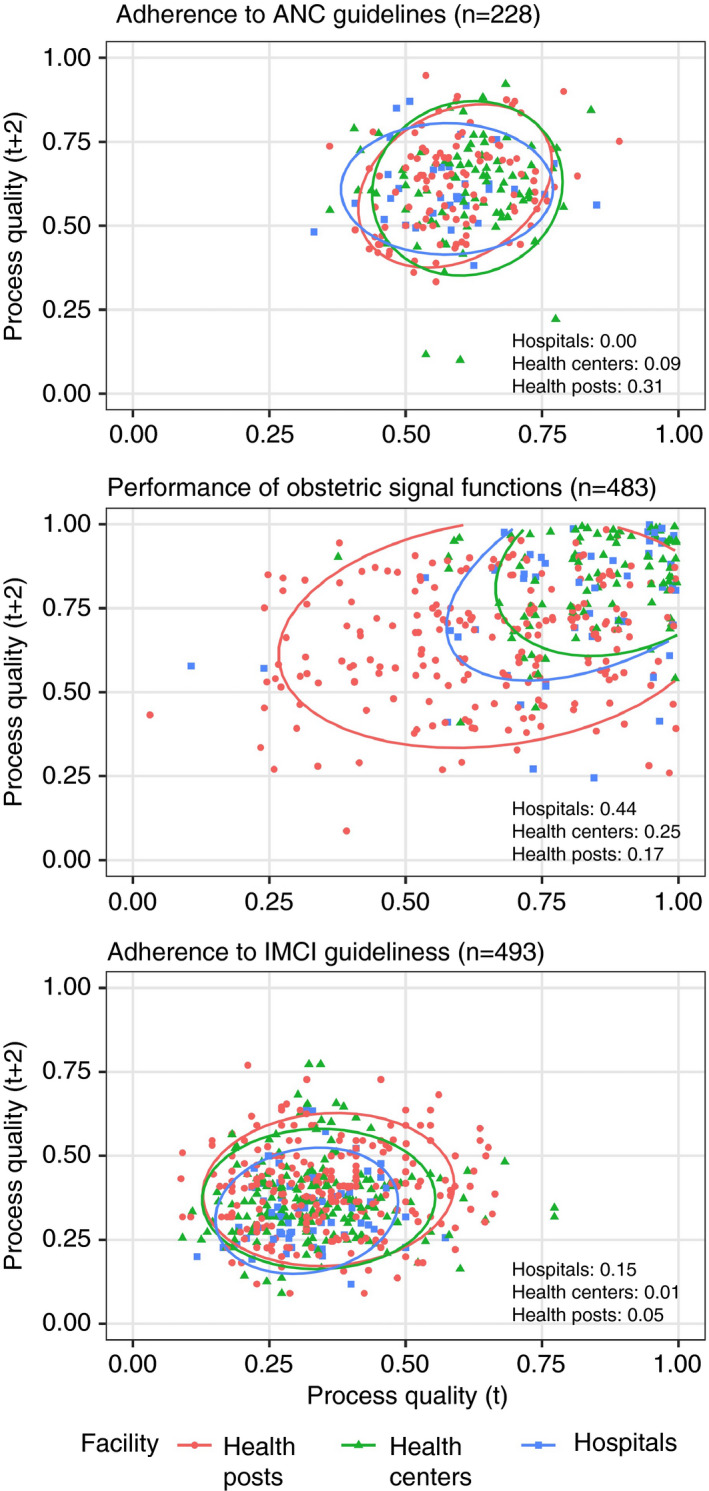
Correlation of process quality measures for individual facilities measured 2 years apart

Process quality measures showed weak or negligible correlation across health facility types except for performance of signal functions in hospitals (Figure 4).

Across all measures, categorical classification showed over 50% agreement only for general and child service readiness and signal functions (Table 3A). Kappa statistics showed ≤33% agreement beyond chance, low even for the most correlated measures.

Results were robust for all measures when using sampling weights from the second wave (Table S6).

### Stability of quality measures within regions

Averaging across facilities with unstable quality measures resulted in relatively small net change in quality measures at the regional level, with the largest differences for adherence to guidelines in sick child care (Figure 2, bottom panel). The positive trends in quality measures are shown in Figures S1 and S2. Structural quality measures were moderately to strongly correlated at the regional level (Table 3B). Adherence to guidelines was strongly correlated for ANC within region despite low correlation at the facility level, but negatively correlated for child services (−0.36). These quality measures were based on similar numbers of facilities and observations within region.

### Impact of instability in quality measures on adjusted coverage estimates

Adjusted coverage could be compared for 28 estimates at the national level (4 years of DHS surveys with matched SPA data for each measure, except process quality of ANC with 2 each). Eighty‐three of the possible 112 regional estimates had ≥1 facility per type and could be included for analysis. Adjusted coverage estimates can be conceptualised as weighted averages of facility quality based on the proportion of those in need choosing that facility type. Health posts were the most commonly used facilities across all services, capturing 71% of ANC 1 users, 66% of ANC 4 users, 56% of deliveries and 80% of sick child visits across the years of DHS surveys. A data file of all adjusted coverage estimates is available as Supplemental Material.

**FIGURE 5:**
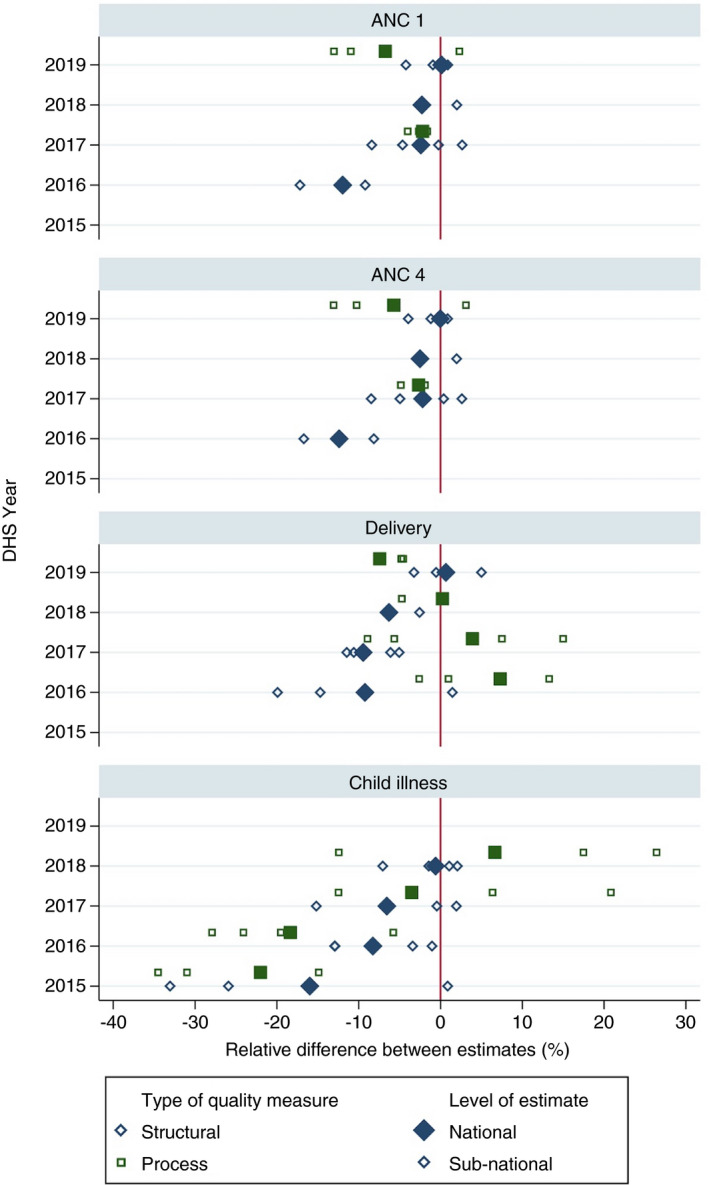
Relative difference in quality‐adjusted coverage estimates using measures of the same facilities 2 years apart

Figure 5 displays the relative difference between adjusted coverage estimates calculated using the reference year SPA and the same facilities assessed 2 years prior. At the national level, the median relative difference in adjusted coverage estimates across years and services was 6.0%, corresponding to a linear difference of 1.9 percentage points (pp). Sub‐national differences were more variable (Table 4). As expected given that adjusted coverage is the product of contact coverage and quality, linear differences were larger in services with higher contact coverage (ANC 1 and delivery). Using relative difference as a common metric across services, quality‐adjusted coverage was less or as divergent than input‐adjusted coverage for ANC and more divergent for delivery. Quality‐adjusted coverage was much more divergent than input‐adjusted coverage for child health services: median relative difference of 19.6% compared to 2.7%, linear differences of 2.4 and 0.7 pp respectively.

**TABLE 4:** Coverage estimates, quality‐adjusted coverage estimates and absolute value of differences in quality‐adjusted coverage estimates for sub‐national regions. Adjusted coverage, SPA_t_ refers to estimates based on the assessment during the reference year SPA for facilities also assessed 2 years prior. Contact coverage and adjusted coverage estimates are provided to aid interpretation of difference, not as generalizable estimates

Contact coverage measure			Coverage	Adjusted coverage, SPA_t_	Absolute value of linear difference in estimates	Absolute value of relative difference in estimates (%)
	Quality measure	*N*	Median (IQR)	Median (IQR)	Median (IQR)	Median (IQR)
At least 1 antenatal care visit (ANC 1)	Service readiness	11	94.4 (91.8, 96.3)	68.2 (63.0, 70.1)	3.3 (0.7, 6.2)	4.2 (0.9, 9.2)
	Adherence to guidelines	6	93.7 (91.8, 96.6)	56.4 (55.6, 58.8)	1.9 (1.3, 7.1)	3.4 (2.3, 11)
At least 4 antenatal care visits (ANC 4)	Service readiness	11	50.9 (48, 62.2)	38.4 (34.9, 40.1)	1.6 (0.5, 3.3)	4.0 (1.2, 8.5)
	Adherence to guidelines	6	50.4 (48, 62.2)	30.9 (29.9, 37.8)	1.5 (0.8, 3.4)	4.0 (2.9, 10.3)
Delivery at a health facility	Service readiness	11	74.5 (67.2, 90.9)	45.1 (41.9, 52.8)	2.3 (1.1, 5.7)	5.1 (2.6, 11.5)
	Obstetric signal functions	11	74.5 (67.2, 90.9)	56.2 (45.5, 63.4)	3.0 (2.2, 5.2)	5.7 (4.6, 9.0)
Child with symptom brought to a health facility	Service readiness	14	36.2 (31.9, 42.3)	24.4 (20.3, 26.8)	0.7 (0.3, 3.5)	2.7 (1.1, 12.9)
	Adherence to guidelines	13	36.4 (31.9, 42.3)	12.8 (10.4, 15.5)	2.4 (2.0, 3.8)	19.6 (12.5, 26.4)

## DISCUSSION

We used the continuous health facility assessments in Senegal to test the stability of quality measures over 2 years for use at the facility level, regional level and in adjusted coverage calculations. We found substantial change in measures of quality of care over 2 years within overall positive trends. Facility measures of structural quality and on performance of BEmONC signal functions were moderately stable as continuous scores, particularly among hospitals. Direct observation of primary care did not provide stable information on level or classification of facility quality. Regional quality measures were at least moderately stable for structural quality and direct observation of ANC. Instability in quality estimates resulted in a median relative difference of ±6% across national adjusted coverage estimates and as much as 20% for regional estimates of adjusted coverage based on direct observation of child services. Careful consideration is required of the types of measure, facility and summary method in using health facility assessment information more than a year after data collection, with generally greater stability in structural measures and in aggregate measures pooling across multiple facilities.

Facility‐level findings suggest a hierarchy of measures in terms of consistency: general and child service readiness were at least moderately consistent in all facility types as continuous measures for description/benchmarking; ANC service readiness, basic obstetric service readiness and reported performance of obstetric signal functions were moderately consistent only in hospitals; adherence to guidelines in ANC and sick child care were not consistent at any level of facility. Kappa statistics were low for all measures: classification can exacerbate error in underlying measures [40] and may be unadvisable in this case. Measures of service readiness are based on tangible attributes; they showed greater consistency in hospitals, where baseline readiness tends to be higher and supply chains may be stronger [41]. Greater stability in measures of child service readiness may relate to the measure including more items and showing a broader span of performance at each assessment point. For reported obstetric signal functions, higher service volume in hospitals likely contributes to greater stability [42]. In contrast, measures of process quality reflect the actions of individual providers seeing a small number of distinct patients for each facility, introducing sampling and measurement error [26]. Prior research in Senegal has shown that direct observation shows high reliability between raters assessing the same visit [43]. The magnitude of observer effects in a large study like the SPA has not been quantified, although extensive training to standardise across observations and observers to minimise this effect is a key component of all DHS Program surveys [31]. Nonetheless, our findings provide minimal evidence of direct observations detecting any role of facilities in ensuring that providers deliver minimum expected actions consistently in essential primary care.

For most measures, facility instability averaged out within regions, resulting in strong or moderate correlation of regional measures over time. The exceptions were obstetric signal functions and direct observation of sick child care, which was negatively correlated despite being based on similar numbers of providers and patient visits as for ANC. Prior research has also found unexplained variation in direct observation of child visits [26]; these findings call into question the value of this assessment as currently conducted.

The assessment of adjusted coverage in this work provides an empirical estimate for the expected measurement error when using an older health facility assessment to link with population data in settings like Senegal. (We do not provide nationally representative estimates of adjusted coverage such as can be found elsewhere [38]). We found relative differences of 6% on average nationally and 3% to 20% within large subnational regions. Discrepancies related to process quality for child health services were by far the largest. These differences corresponded to plus or minus 1 to 3 percentage points difference between estimates in most cases; whether this discrepancy is meaningful may depend on the context of the research or policy in question. The relative difference in adjusted coverage estimates was above 5% for measures with the weakest correlation among commonly used facilities (health posts): structure and process measures for delivery care and direct observation of sick child visits. Differences in adjusted coverage will be largest when contact coverage is high, correlation is weak in the most‐used facilities and number of facilities per stratum is small. These elements should be considered in generalising to other settings; for instance, estimates may be more stable in settings or services where higher level facilities are more frequently used. Consistent inclusion of confidence intervals to capture at least sampling error [37, 38] and acknowledgement of potential measurement error is warranted for adjusted coverage calculations extrapolating over time.

Aspects of this study design should be considered in extrapolating the findings. This study was conducted on the subset of facilities that could be linked over a 2‐year period in Senegal from 2012/2013 to 2018, which included most hospitals and health centres included in the SPA and a subset of health posts (no health huts). The findings should be generalisable to facilities of these types within Senegal and potentially in similar health systems, particularly within health facility tier and for higher level facilities better represented in this study. Findings on adjusted coverage are limited to the particulars of these data: simulation studies would be needed to further detail expected differences for a range of sample sizes and levels of stability. Due to the nature of the resampling, we are not able to assess stability of measures over a shorter duration or to consider all facilities over 3 or more surveys. Application of these findings to other settings or times must also consider potential external changes to health services, whether a policy reform or a systemic shock such as the Covid‐19 pandemic, that may produce much greater change than observed here.

Health facility assessments conducted in Senegal have yielded a range of important insights into the health system and the quality of maternal and child health services [44, 45, 46, 47]. Despite the strong national policy towards realisation of UHC, however, these assessments have not been heavily integrated within national policy documents and monitoring strategies in Senegal [28]. As in many countries, health system managers and policymakers face a proliferation of data sources without clear evidence on suitable, efficient and reliable sources of information to inform action. The lack of a predefined plan for data use for policy and research has been identified as a limitation of health facility assessments like SPA to date [48]. The unique nature of the continuous SPA enables consideration of how frequently such assessments would need to be conducted to provide stable insight on performance of facilities or regions. This evidence should be incorporated within national priorities for data use and action to shape health system measurement. For instance, while structural quality measures showed greater stability, if their intended purpose is to lead to corrective action, routine monitoring would better support timely response. Measures that are stable but not useful provide little value for cost. Whether the differences identified in adjusted coverage estimates are meaningful enough to increase the frequency of measurement depends on the acceptable degree of error (measurement as well as sampling) for a given application; they should certainly be considered when reporting any adjusted coverage analyses. Direct observation is a powerful method of assessing quality with clear benefits relative to routine indicators or record review [49], but its use for assessing sick child services requires further consideration to determine if this measure can provide reliable information within any timeframe with current sample sizes. Targeting direct observation appropriately and deploying it strategically would enhance the value of this approach. Priority changes include incorporating clinical indicators like correct diagnosis and treatment and better defining the minimum number of direct observations required to reliably support the intended use of resulting indicators. Using tools like health facility assessments sparingly but appropriately is one step towards more actionable and less cumbersome insights into quality of care.

## CONFLICT OF INTERESTS

Dr. Leslie discloses research funding from ICF International, the World Bank, the World Health Organization and the US National Institutes of Health unrelated to the conduct of this research. Dr. Nimako reports being a consultant for the Bill & Melinda Gates Foundation during the conduct of the study. The authors declare no conflicts of interest.

## Supporting information

Supplementary Material

Supplementary Material

Appendix

## Data Availability

Data for this analysis are publicly available from The DHS Program (dhsprogram.com) and on request from ANSD (statsenegal@ansd.sn). Programming code is available on request from the corresponding author and at https://osf.io/y5n87/. Adjusted coverage estimates are included as Supplemental Material.
