# Supplementary material for: Stability of healthcare quality measures for maternal and child services: Analysis of the continuous service provision assessment of health facilities in Senegal, 2012–2018
Source: Trop Med Int Health. 2021 Dec 16;27(1):68–80. doi: 10.1111/tmi.13701 (PMC9300084; doi:10.1111/tmi.13701)

**Supplemental Materials**

**Table S1. Quality Measure Summary**

| **Service** | **Structural quality (inputs to care) measure** | **Process quality measure** |
| --- | --- | --- |
| General | WHO general service readiness index | None |
| Maternal: antenatal care | WHO service readiness index for antenatal care | Adherence to the focused ANC model during ANC visits |
| Maternal: obstetric care | WHO service readiness index for basic obstetric care | Performance of basic obstetric signal functions in past 3 months |
| Child: curative care | WHO service readiness index for child health (preventive & curative) | Adherence to IMCI during sick child visits |

**Measure details**

**General service readiness**

Average of the proportion of items present in the 5 domains below

*Basic amenities*

| - Power (electricity for lights and communication from any power source with break less than 2hours/per day) - Improved water source within 500 m of facility - Room with auditory and visual privacy for patient consultations | - Access to adequate sanitation facilities for clients - Communication equipment (phone or short-wave radio) - Access to computer with e-mail and Internet - Emergency transportation |
| --- | --- |

*Basic equipment*

| - Adult scale - Child scale - Thermometer | - Stethoscope - Blood pressure apparatus - Light source |
| --- | --- |

*Infection prevention*

| - Safe final disposal of sharps - Safe final disposal of infectious wastes - Appropriate storage of sharps waste (sharps box/container) - Appropriate storage of infectious waste (waste receptacle with lid and plastic bin liner) | - Disinfectant - Single-use, standard disposable or auto-disable syringes - Soap and running water or alcohol-based hand rub - Latex gloves - Guidelines for standard precautions |
| --- | --- |

*Diagnostic capacity*

| - Haemoglobin - Blood glucose - Malaria diagnostic capacity - Urine dipstick - protein | - Urine dipstick - glucose - HIV diagnostic capacity - Syphilis RDT - Urine pregnancy test |
| --- | --- |

*Medication*

| - Amitriptyline tablet - Amlodipine tablet or alternative calcium channel blocker - Amoxicillin syrup/suspension or dispersible tablet - Amoxicillin tablet - Ampicillin powder for injection - Beclometasone inhaler - Ceftriaxone injection - Enalapril tablet or alternative ACE inhibitor e.g. lisinopril, ramipril, perindopril - Gentamicin injection | - Glibenclamide tablet - Ibuprofen tablet - Insulin injection - Metformin tablet - Omeprazole tablet or alternative such as pantoprazole, rabeprazole - Oral rehydration solution - Paracetamol tablet - Salbutamol inhaler - Simvastatin tablet or other statin e.g. atorvastatin, pravastatin, fluvastatin - Zinc sulphate tablet or syrup |
| --- | --- |

**Antenatal care**

Service readiness

| - Guidelines on ANC - Staff trained in ANC - Blood pressure apparatus - Haemoglobin - Urine dipstick- protein | - Iron Tablets - Folic Acid Tablets - Tetanus Toxoid Vaccine - IPT Drug - ITNs |
| --- | --- |

Adherence to guidelines

| *Quality defined as the percent of the following items performed. Items recommended once or twice during three follow-up visits are down-weighted by one third and two thirds respectively* | First visit | Follow up visits |
| --- | --- | --- |
|  |  |  |
| *History Taking - Provider asked or checked:* |  |  |
| - Last menstrual period to calculate gestational age | 1 |  |
| - Prior pregnancy experience, e.g. at least one of stillbirth, neonatal death, heavy bleeding, assisted delivery, multiples, and spontaneous or assisted abortion | 1 |  |
| - Danger signs in current pregnancy, e.g. at least one of bleeding, fever, headache or blurred vision, swollen face or hands, tiredness or breathlessness, fetal movements | 1 | 1 |
| - Previous complications on record |  | 1 |
| *Routine Examination – Provider performed, examined for or assessed:* | |  |
| - Weight | 1 | 1 |
| - Fundal height | 1 | 1 |
| - Edema | 1 | 1 |
| - Vaginal exam | 1 |  |
| - Blood pressure | 1 | 1 |
| - Fetal heart rate |  | 1 |
| - Ultrasound | 1 |  |
| *Screening – Provider asked about, performed, or referred patient for:* |  |  |
| - Syphilis test | 1 | 1/3 |
| - HIV testing and counseling | 1 |  |
| - Anemia test | 1 | 2/3 |
| - Blood group test | 1 |  |
| - Urine test (proteinuria, bacteriuria) | 1 | 2/3 |
| *Preventative measures* |  |  |
| - Prescribed or gave iron or folic acid or both | 1 | 1 |
| - Provider prescribed or gave tetanus toxoid injection | 1 | 1/3 |
| - Prescribed or gave intermittent preventive treatment in pregnancy (IPTp) | | 1 |
| *Education – Provider counseled on:* |  |  |
| - Nutrition | 1 | 1 |
| - Sleeping under an insecticide-treated net | 1 | 1 |
| - Delivery planning: preparation (money, transport) and location | 1 | 1 |
| - Emergency planning: supplies for home delivery | 1 | 1 |
| - Breastfeeding |  | 2/3 |
| - Post-partum and postnatal care |  | 2/3 |
| - Pregnancy spacing |  | 2/3 |
| *Record keeping* |  |  |
| - Provider completed ANC card | 1 | 1 |
|  |  |  |

**Obstetric care**

Service readiness

| - Guidelines for Integrated management of pregnancy and childbirth (IMPAC) - Staff trained in IMPAC - Emergency transport - Sterilization equipment - Examination light - Delivery pack - Suction apparatus (mucus extractor) - Manual vacuum extractor - Vacuum aspirator or D&C kit | - Neonatal bag and mask - Delivery bed - Partograph - Gloves - Antibiotic Eye Ointment for newborn - Injectable uterotonic - Injectable antibiotic - Injectable magnesium sulphate - Skin disinfectant - IV solution with infusion set |
| --- | --- |

Facility reports performance of the following basic signal functions in past 3 months

| - Assisted vaginal delivery - Remove retained products of conception - Administered parenteral oxytocin for haemorrhage - Parenteral magnesium sulphate for (pre-)eclampsia | - Manual removal of placenta - Antibiotics for maternal infection - Neonatal resuscitation using bag and mask |
| --- | --- |

**Sick child care**

Service readiness

| - Guidelines for IMCI - Guidelines for growth monitoring - Staff trained in IMCI - Staff trained in growth monitoring - Child and infant scale - Length/height measuring equipment - Thermometer - Stethoscope - Growth Chart - Haemoglobin | - Test Parasite in Stool (microscopy) - Malaria Diagnostic Capacity - Oral Rehydration Solution Packet - Amoxicillin tablet or syrup - Co-trimoxazole syrup/suspension - Paracetamol syrup/suspension - Vitamin A capsules - Me-/albendazole cap/tab - Zinc sulphate tablets or syrup |
| --- | --- |

Adherence to IMCI protocol

Average of the items relevant for visits for infants <2 months and for children 2 – 59 months

|  | < 2 months | 2 - 59 months |
| --- | --- | --- |
| *History taking - Provider asks:* |  |  |
| - Inability to drink anything | 1 | 1 |
| - Normal feeding pattern | 1 | 0 |
| - Sick feeding pattern | 1 | 0 |
| - Cough or difficult breathing | 0 | 1 |
| - Diarrhoea and blood in stool (dysentery) | 1 | 1 |
| - Fever | 0 | 1 |
| - Vomiting | 0 | 1 |
| - Convulsions | 1 | 1 |
| - Maternal HIV status | 1 | 1 |
| - Ear problems | 0 | 1 |
| *Routine Examination* |  |  |
| - Weight | 1 | 1 |
| - Plotted weight on chart | 1 | 1 |
| - Temperature | 1 | 1 |
| - Pallor | 0 | 1 |
| - Oedema of feet | 0 | 1 |
| - Count respirations | 1 | 1 |
| - Mouth (thrush in IMCI) | 1 | 0 |
| *Drug Administration and Immunization* |  |  |
| - Immunized during visit/Checks Immunization Card | 1 | 1 |
| - Vitamin A dosage | 1 | 1 |
| - Deworming medication | 0 | 1 |
| *Client Education and Counselling* |  |  |
| - Explains how to administer prescribed medication | 1 | 1 |
| - Directions for feeding | 1 | 1 |
| - Describes danger signs requiring return to facility | 1 | 1 |
| - Scheduled/discussed return visit | 1 | 1 |
| - Gave diagnosis | 0 | 1 |
|  |  |  |

**Table S2: Differences calculated for adjusted coverage estimates**

| Adjusted coverage estimate based on analytic sample in reference SPA survey | ${adj cov}_{SPA t}$ |
| --- | --- |
| Adjusted coverage estimate based on analytic sample in SPA survey 2 years prior to reference survey | ${adj cov}_{SPA t-2}$ |
| Difference in adjusted coverage estimates (linear percentage points) | ${adj cov}_{SPA t-2}-{adj cov}_{SPA t}$ |
| Absolute value of difference in adjusted coverage | $abs\left( {adj cov}_{SPA t-2}-{adj cov}_{SPA t} \right)$ |
| Relative difference in adjusted coverage estimates (percent) | $\left( \frac{\left( {adj cov}_{SPA t-2 years}-{adj cov}_{SPA t} \right)}{{adj cov}_{SPA t}} \right)*100$ |
| Absolute value of relative difference | $abs\left( \frac{\left( {adj cov}_{SPA t-2 years}-{adj cov}_{SPA t} \right)}{{adj cov}_{SPA t}} \right)*100$ |

**Table S3: Characteristics of hospitals, health centers, and health posts sampled for assessment in Senegal, 2012 – 2018, Unweighted**

| \|  \| 2012 and 2013 \| 2014 \| 2015 \| 2016 \| 2017 \| 2018 \| \| --- \| --- \| --- \| --- \| --- \| --- \| --- \| \|  \| (N = 364) \| (N = 363) \| (N = 375) \| (N = 371) \| (N = 396) \| (N = 339) \| |
| --- | --- | --- | --- | --- | --- | --- | --- | --- | --- | --- | --- | --- | --- | --- |
| \| **Facility type** \|  \|  \|  \|  \|  \|  \| \| --- \| --- \| --- \| --- \| --- \| --- \| --- \| \| Hospital \| 35 (10%) \| 35 (10%) \| 38 (10%) \| 35 (9%) \| 35 (9%) \| 29 (9%) \| \| Health center \| 64 (18%) \| 62 (17%) \| 65 (17%) \| 61 (16%) \| 74 (19%) \| 62 (18%) \| \| Health post \| 265 (73%) \| 266 (73%) \| 272 (73%) \| 275 (74%) \| 287 (72%) \| 248 (73%) \| \| **Facility location** \|  \|  \|  \|  \|  \|  \| \| Rural \| 196 (54%) \| 205 (56%) \| 198 (53%) \| 201 (54%) \| 238 (60%) \| 7 (2%) \| \| Urban \| 168 (46%) \| 158 (44%) \| 177 (47%) \| 170 (46%) \| 158 (40%) \| 332 (98%)* \| \| **Facility managing authority** \|  \|  \|  \|  \|  \|  \| \| Public \| 305 (84%) \| 293 (81%) \| 299 (80%) \| 288 (78%) \| 315 (80%) \| 270 (80%) \| \| Private \| 59 (16%) \| 70 (19%) \| 76 (20%) \| 83 (22%) \| 81 (20%) \| 69 (20%) \| \| **Services provided** \|  \|  \|  \|  \|  \|  \| \| ANC \| 319 (88%) \| 326 (90%) \| 327 (87%) \| 323 (87%) \| 343 (87%) \| 299 (88%) \| \| Delivery \| 281 (77%) \| 282 (78%) \| 286 (76%) \| 301 (81%) \| 315 (80%) \| 269 (79%) \| \| Curative care for sick children \| 342 (94%) \| 349 (96%) \| 356 (95%) \| 356 (96%) \| 372 (94%) \| 329 (97%) \| \| **Facilities with direct observation** \|  \|  \|  \|  \|  \|  \| \| ANC (N=1937)† \| 0 (0%) \| 300 (92%) \| 0 (0%) \| 290 (90%) \| 0 (0%) \| 206 (69%) \| \| Curative care of sick children (N=2104) \| 327 (96%) \| 316 (91%) \| 329 (92%) \| 313 (88%) \| 321 (86%) \| 221 (67%) \| |

*The definition of rural vs. urban setting changed before the 2018 assessment such that many facilities classified as rural in prior years were considered urban in 2018; the sampling approach, which is independent of urban/rural location, did not change.

† Observations of ANC visits conducted every other year.

**Table S4: Two-year difference in quality measures by facility type, weighted by first wave sampling weight**

|  | Hospital | Health center | Health post | Total |
| --- | --- | --- | --- | --- |
|  | (N = 104) | (N = 211) | (N = 313) | (N = 628) |
|  | Mean (SD) | Mean (SD) | Mean (SD) | Mean (SD) |
| **Service readiness** |  |  |  |  |
| Absolute difference | 0.07 (0.06) | 0.07 (0.06) | 0.09 (0.07) | 0.08 (0.07) |
| Net difference | 0.01 (0.09) | 0.03 (0.09) | 0.05 (0.10) | 0.04 (0.10) |
| N (%) | 104 (100.0%) | 211 (100.0%) | 313 (100.0%) | 628 (100.0%) |
| **ANC service readiness** |  |  |  |  |
| Absolute difference | 0.12 (0.11) | 0.13 (0.11) | 0.13 (0.11) | 0.13 (0.11) |
| Net difference | 0.00 (0.16) | 0.02 (0.17) | 0.03 (0.17) | 0.03 (0.17) |
| N (%) | 83 (79.8%) | 164 (77.7%) | 275 (87.9%) | 522 (83.1%) |
| **Basic obstetrics readiness** |  |  |  |  |
| Absolute difference | 0.14 (0.13) | 0.15 (0.12) | 0.17 (0.13) | 0.16 (0.13) |
| Net difference | -0.01 (0.19) | 0.03 (0.19) | 0.04 (0.21) | 0.03 (0.20) |
| N (%) | 85 (81.7%) | 147 (69.7%) | 251 (80.2%) | 483 (76.9%) |
| **Child service readiness** |  |  |  |  |
| Absolute difference | 0.12 (0.09) | 0.12 (0.09) | 0.11 (0.09) | 0.11 (0.09) |
| Net difference | 0.03 (0.15) | 0.04 (0.15) | 0.05 (0.13) | 0.05 (0.14) |
| N (%) | 87 (83.7%) | 198 (93.8%) | 298 (95.2%) | 583 (92.8%) |
| **Adherence to ANC guidelines** | |  |  |  |
| Absolute difference | 0.11 (0.10) | 0.13 (0.10) | 0.11 (0.08) | 0.12 (0.09) |
| Net difference | 0.04 (0.15) | 0.01 (0.16) | 0.02 (0.14) | 0.02 (0.15) |
| N (%) | 33 (31.7%) | 82 (38.9%) | 113 (36.1%) | 228 (36.3%) |
| **Signal functions (of 7)** |  |  |  |  |
| Absolute difference | 0.12 (0.14) | 0.12 (0.12) | 0.21 (0.17) | 0.18 (0.16) |
| Net difference | -0.02 (0.18) | -0.02 (0.17) | 0.01 (0.26) | 0.00 (0.24) |
| N (%) | 85 (81.7%) | 147 (69.7%) | 251 (80.2%) | 483 (76.9%) |
| **Adherence to sick child guidelines** | |  |  |  |
| Absolute difference | 0.10 (0.09) | 0.13 (0.11) | 0.14 (0.11) | 0.14 (0.11) |
| Net difference | 0.02 (0.13) | 0.03 (0.17) | 0.04 (0.18) | 0.04 (0.17) |
| N (%) | 66 (63.5%) | 175 (82.9%) | 252 (80.5%) | 493 (78.5%) |

**Table S5: Pairwise correlation of domains of service readiness over 2 years, weighted by first wave sampling weight**

| **General** | N | Basic amenities | Staff | Basic equipment | Infection prevention | Diagnostics | Medications |
| --- | --- | --- | --- | --- | --- | --- | --- |
| All facilities | 628 | 0.68 | NA | 0.33 | 0.28 | 0.39 | 0.56 |
| Hospitals | 104 | 0.59 | NA | 0.56 | 0.46 | 0.45 | 0.70 |
| Health centers | 211 | 0.63 | NA | 0.36 | 0.44 | 0.46 | 0.48 |
| Health posts | 313 | 0.48 | NA | 0.28 | 0.21 | 0.38 | 0.52 |
| **ANC** |  |  |  |  |  |  |  |
| All facilities | 522 | NA | 0.17 | -0.01 | NA | 0.29 | 0.43 |
| **Basic obstetrics** |  |  |  |  |  |  |  |
| All facilities | 483 | NA | 0.20 | 0.52 | NA | NA | 0.24 |
| **Child services** |  |  |  |  |  |  |  |
| All facilities | 583 | NA | 0.41 | 0.54 | NA | 0.69 | 0.56 |

**Table S6: Main findings on correlation of facility-level quality measures using sampling weight from the second time facilities were assessed**

|  | Correlation | | | |
| --- | --- | --- | --- | --- |
| *Service readiness measures* | Overall | Hospitals | Health centers | Health posts |
| General | 0.61 | 0.73 | 0.77 | 0.50 |
| Antenatal care | 0.36 | 0.62 | 0.25 | 0.32 |
| Basic obstetrics | 0.25 | 0.43 | 0.30 | 0.17 |
| Care for children | 0.65 | 0.77 | 0.73 | 0.56 |
| *Process quality measures* |  |  |  |  |
| Antenatal care | 0.24 | 0.11 | 0.07 | 0.30 |
| Basic obstetrics | 0.23 | 0.47 | 0.20 | 0.03 |
| Care for children | 0.02 | 0.15 | 0.01 | 0.00 |

**Supplemental Figure S1: Levels of Structural Quality, 2012 – 2018**

Regional and national summaries based only on facilities assessed at least twice across the 6 years of assessment


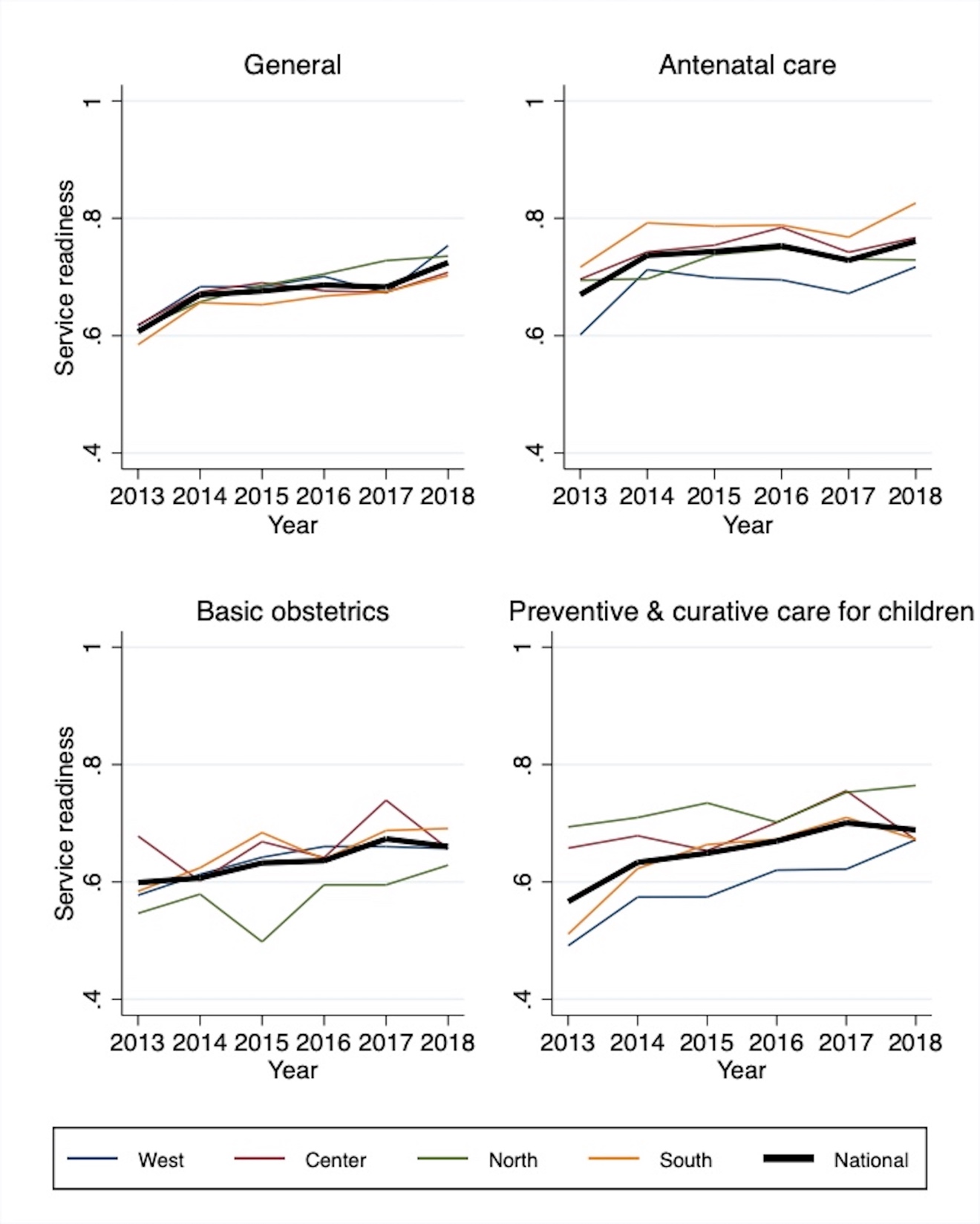


**Supplemental Figure S2: Levels of Process Quality, 2012 – 2018**

Regional and national summaries based only on facilities assessed at least twice across the 6 years of assessment


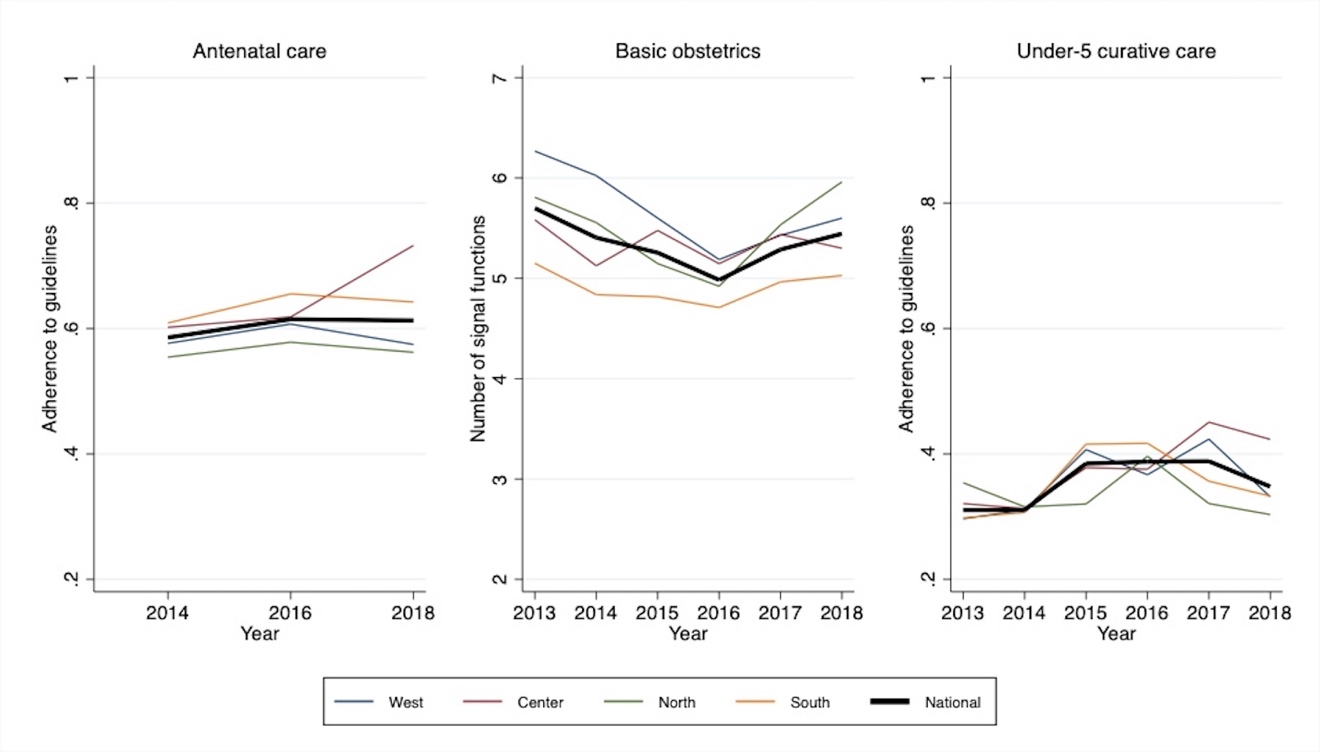

Supplement: Supplementary file 1 — Supplementary Material [file TMI-27-68-s002.docx]
